# Palliative chemotherapy for pancreatic adenocarcinoma: a retrospective cohort analysis of efficacy and toxicity of the FOLFIRINOX regimen focusing on the older patient

**DOI:** 10.1186/s12876-017-0709-3

**Published:** 2017-12-06

**Authors:** Anne Katrin Berger, Georg Martin Haag, Martin Ehmann, Anne Byl, Dirk Jäger, Christoph Springfeld

**Affiliations:** 1 Department of Medical Oncology, National Center for Tumor Diseases (NCT), Heidelberg University Hospital, Im Neuenheimer Feld 460, 69120 Heidelberg, Germany; 2 Pharmacy Department, Heidelberg University Hospital, Heidelberg, Germany; 3 NCT Clinical Cancer Registry, German Cancer Research Center, Heidelberg, Germany

**Keywords:** Pancreatic cancer, Elderly patients, Folfirinox, Toxicity

## Abstract

**Background:**

Pancreatic cancer occurs more frequently in older patients, but these are underrepresented in the phase III clinical studies that established the current treatment standards. This leads to uncertainty regarding the treatment of older patients with potentially toxic but active regimens like FOLFIRINOX.

**Methods:**

We conducted a retrospective analysis of patients treated according to the FOLFIRINOX protocol at our institution between 2010 and 2014 with a focus on older patients.

**Results:**

Overall survival in our cohort was 10.2 months. Only 43% of patients did not need dose adaptations, but dose reductions did not lead to an inferior survival. We did not find evidence that patients aged 65 years and older deemed fit enough for palliative treatment had more toxicities or a worse outcome than younger patients.

**Conclusion:**

We conclude that treatment with the FOLFIRINOX protocol in patients with pancreatic cancer should not be withhold from patients solely based on their chronological age but rather be based on the patient’s performance status and comorbidities.

## Background

Cancer occurs more frequently in elderly people, and the current demographic changes with an aging population in Western countries will therefore result in a rising incidence of cancer and an increasing amount of older cancer patients [1–3]. For the US, it has been estimated that by 2030, approximately 70% of all cancers will be diagnosed in adults older than 65 years [4]. In contrast to this development, older patients remain underrepresented in the clinical cancer studies that establish standard treatment regimens [5]. There is widespread critique concerning this issue, but to date, trial results have to be extrapolated to the older population, although this approach remains questionable [6]. Fear of increased toxicities and uncertainty concerning both clinical treatment benefit and the patient’s physical resources may cause limitation of tumor-specific therapies in older patients. Ensuring an adequate antitumor treatment while avoiding toxicity is a challenging task for geriatric oncology in daily routine.

For pancreatic adenocarcinoma, the fourth common cause of cancer-related death in the US [7], approximately two-thirds of cases are diagnosed in patients over 65 years [8]. The overall 5-year survival rate is about 6% and remains the poorest of all major malignancies [9, 10]. Because the majority of tumors is irresectable or recurs after surgery, systemic palliative treatment is needed for almost every patient [11]. Despite its limited activity, single-agent gemcitabine was the standard palliative first-line treatment for patients with advanced disease for more than a decade [12], until therapy options improved in 2011. In the landmark PRODIGE 4 trial, the FOLFIRINOX protocol had an impressive response rate of 31.6%, and it significantly improved median overall survival (OS) from 6.8 months in the gemcitabine monotherapy arm to 11.1 months [13]. More recently, this protocol has also been used successfully in patients with irresectable, locally advanced disease to achieve resectability and therefore offering a chance for cure [14]. The impressive results of the FOLFIRINOX protocol are accompanied by significantly increased grade 3 and 4 toxicities, mainly myelosuppression, diarrhea and peripheral neuropathy. Concerns regarding safety in the palliative setting have been raised immediately [15]. Likewise, the study population was criticized as heavily selected (young age, excellent performance status, mostly “non-head” tumors), not representing the average “real-life” patient. Subsequent retrospective clinical analyses confirmed the substantial toxicity profile, and modifications of the regimen are commonly recommended [16–18]. In 2013, therapeutic options further increased with publication of the MPACT trial. In this study, the addition of nab-paclitaxel to gemcitabine therapy increased the median overall survival from 6.6 to 8.7 months [19].

Thus, oncologists might be reluctant to apply FOLFIRINOX to older patients. Given the undoubted advantages in response rate and survival, older cancer patients might be at risk for therapeutic disparity and undertreatment. We conducted a retrospective analysis of patients with advanced pancreatic cancer under palliative first-line treatment with FOLFIRINOX at the National Center for Tumor Diseases, Heidelberg, to assess efficacy and toxicity in academic practice, especially focusing on older patients.

## Methods

### Patients

Requirements for inclusion were (1) histologically proven diagnosis of ductal pancreatic adenocarcinoma, (2) irresectable (metastasized or locally advanced) disease and (3) palliative first-line treatment with FOLFIRINOX at the NCT Heidelberg, Germany between January 2010 and June 2014. The observation period for each patient started with initiation of first-line treatment (i.e. first systemic chemotherapy after primary diagnosis of metastatic or inoperable disease or, in resected patients, after diagnosis of recurrence**).** The follow-up period for this analysis ended on July, 15th 2015. Survival data were available for all patients. The patients were identified with permission of two own institutional databases (the *NCT clinical cancer registry, a* prospectively maintained database and the registry of the pharmacy department of the University hospital Heidelberg, respectively).

### Treatment

Full dose FOLFIRINOX consisted of oxaliplatin 85 mg/m^2^, irinotecan 180 mg/m^2^, leucovorin 400 mg/m^2^, fluorouracil 400 mg/m^2^ bolus and 2400 mg/m^2^ over 46 h, q2w, as originally described [13]. Dose modifications were at the discretion of the treating physician.

### Assessment

Clinical data were documented via an electronic medical record system. Information included Eastern Cooperative Oncology Group performance status (ECOG PS) [20], presence and site of metastases at diagnosis, date of previous surgery and adjuvant chemotherapy, start and stop date of FOLFIRINOX treatment, type and severity of toxicities and consecutive dose reductions, response to first-line therapy, date of progression, and date of death. Toxic effects were registered according to the National Cancer Institute’s common terminology criteria for adverse events (CTCAE). Tumor response was routinely evaluated according to the response evaluation criteria in solid tumors (RECIST, [21]).

### Statistical analysis

Man Whitney U-Test and Fisher’s exact test were used for comparing independent samples of quantitative and binary data, respectively. Progression-free survival (PFS) was defined as time from start of palliative first-line treatment to documented tumor progression or death. Overall survival (OS) was defined as the time from start of palliative first-line treatment to death. Time-to event data were analyzed using standard methods, including Kaplan-Meier product-limit estimates. All analyses of prognostic factors were of an exploratory nature. Statistical analysis was performed using the SPSS statistical software, Version 22.

## Results

### Patients’ demographics

We identified 88 patients meeting the inclusion criteria. Median duration of observation was 10.4 months. The median age at diagnosis of advanced disease was 56 years (range 32–78), 15 patients (17%) were 65 years or older, and 8 patients (9%) were ≥70 years. 80 patients (91%) had died at the time of analysis. 50 patients (57%) had pancreatic head tumors, and 79 patients (90%) had metastatic disease. 22 patients (25%) had undergone prior tumor resection, and 13 (15%) had initially received adjuvant chemotherapy. 85 patients (97%) started therapy with an ECOG of 0 or 1. The main characteristics concerning both tumor disease and patient demographics did not differ significantly between younger (< 65 years) and older (≥ 65 years) patients. The patient characteristics are summarized in Table 1.

**Table 1:** Patient characteristics

Patient characteristics	all	<65 years	≥ 65 years
Number of Patients	88	73	15
Median age (range), years	56 (32–78)		
	n (%)		
Gender			
Female	31 (35.2)	29 (39.7)	2 (13.3)
Male	57 (64.8)	44 (60.3)	13 (86.7)
ECOG PS			
0	49 (55.7)	43 (58.9)	6 (40.0)
1	36 (40.9)	27 (37.0)	9 (60.0)
2	3 (3.4)	3 (4.1)	0
Metastatic disease	79 (89.8)	64 (87.7)	15 (100.0)
Locally advanced tumor	9 (10.2)	9 (12.3)	0
Primary palliative treatment	66 (75.0)	55 (75.3)	11 (73.3)
Previous resection	22 (25.0)	18 (24.7)	4 (26.7)
Prior (neo-) adjuvant CTX	13 (14.8)	11 (15.1)	2 (13.3)
Site of Tumor			
Pancreatic head	50 (56.8)	42 (57.5)	8 (53.3)
Pancreatic corpus	22 (25.0)	20 (27.4)	2 (13.3)
Pancreatic tail	16 (18.2)	11 (15.1)	5 (33.3)

### FOLFIRINOX and toxicities

Median duration of first-line therapy with FOLFIRINOX was 150 days (range 14–787). Thirty-eight patients (43%) received therapy per protocol without any modifications during the course of treatment. Forty-six patients (52%) developed side effects that were classified as CTCAE grade 3 or higher: Hematologic side effects were found in 11 patients (13%), and 8 (9%) developed severe peripheral neuropathy. Six patients (7%) suffered from severe diarrhea, fatigue or cholangitis, respectively. Seven patients (8%) stopped therapy due to toxicity. There was no therapy-related death. Modifications of the FOLFIRINOX protocol were necessary in 50 patients. Median time to the first reduction was 74 days (range 0–287) after initiation of therapy. 12 patients (13.6%) had dosage modifications of only oxaliplatin of which 7 totally stopped and 5 continued therapy with 80% dosage. In 7 patients (8.0%), solely irinotecan, and in 5 patients (5.7%) the 5-fluorouracil bolus was dropped. 12 patients (13.6%) had a fixed reduction of all 3 cytotoxic drugs of 75–80% of the per-protocol dosage. 12 patients (13.6%) had dose reduction of varying degrees of two (4 patients) or three components (8 patients).

A summary of CTCAE Grade 3 and 4 toxicities is given in Table 2.

**Table 2:** Grade 3 or 4 toxicity according to the CTCAE (version 4)

	all, n (%)	<65 years	≥ 65 years
Any grade ≥ 3 toxicity	46 (52.3)	41 (56.2)	5 (33.3)
Hematological toxicity grade ≥ 3			
Neutropenia	2 (2.3)	2 (2.7)	0
Febrile neutropenia	1 (1.1)	1 (1.4)	0
Thrombopenia/Anemia	3 (3.4)	3 (4.1)	0
Fatigue	6 (6.8)	4 (5.5)	2 (13.3)
Nausea/Vomiting	3 (3.4)	1 (1.4)	2 (13.3)
Diarrhea	6 (6.8)	5 (6.8)	1 (6.7)
Cholangitis	6 (6.8)	5 (6.8)	1 (6.7)
Thrombosis/Pulmonary embolism	18 (20.5)	17 (23.3)	1 (6.7)
Treatment modifications due to tocixity	50 (56.8)	43 (58.9)	7 (46.7)
Permanent treatment stop due to toxicity	7 (8.0)	4 (5.5)	3 (20.0)

### Progression and survival

**Fig. 1:**
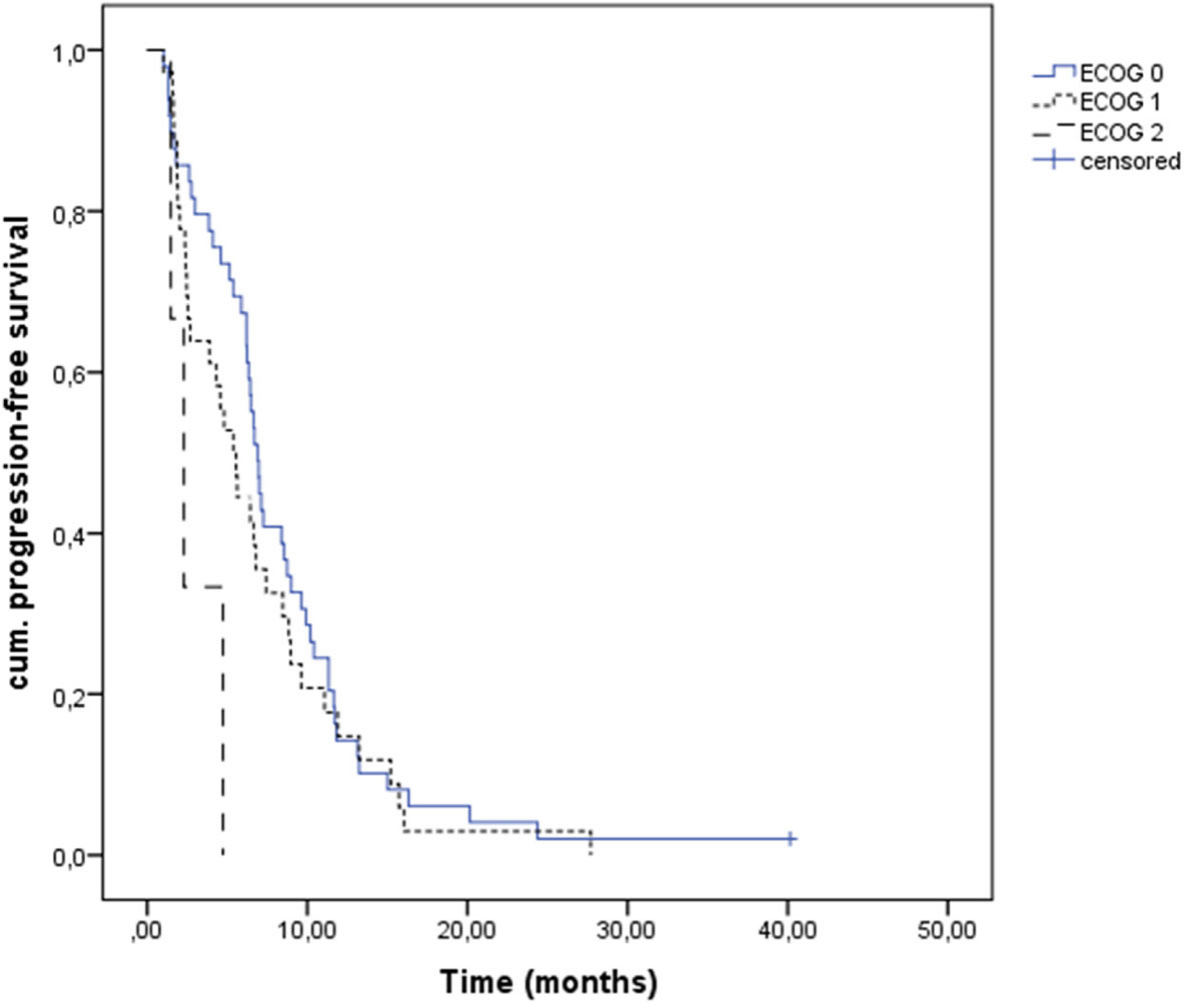
PFS according to ECOG PS

Median PFS of our cohort was 6.4 months [95% CI 5.7;7.2]. It differed significantly between the ECOG groups: it was 6.9 months [95% CI 6.2;7.6] for patients with an ECOG PS 0, 5.4 months [95% CI 3.8;6.9] for ECOG PS 1 and 2.3 months [95% CI 1.0;3.6] for ECOG PS 2 (overall comparison *p* = 0.019) (Fig. 1). Patients needing dose reductions had a longer median PFS than those in the per-protocol group (7.4 months [95% CI 5.6;9.2] vs. 3.8 months [95% CI 0.9;6.8]; *p* = 0.003), however duration of therapy was significantly longer in this group: 180 days vs. 59 days (*p* < 0.001). Patients with therapy discontinuation due to toxicity had a significantly shorter PFS (2.5 months [95% CI 1.3;3.8] vs. 6.7 months [95% CI 6.0;7.4] *p* = 0.01). There were no apparent PFS associations for metastasized compared to locally advanced tumors or for different tumor localizations.

**Fig. 2:**
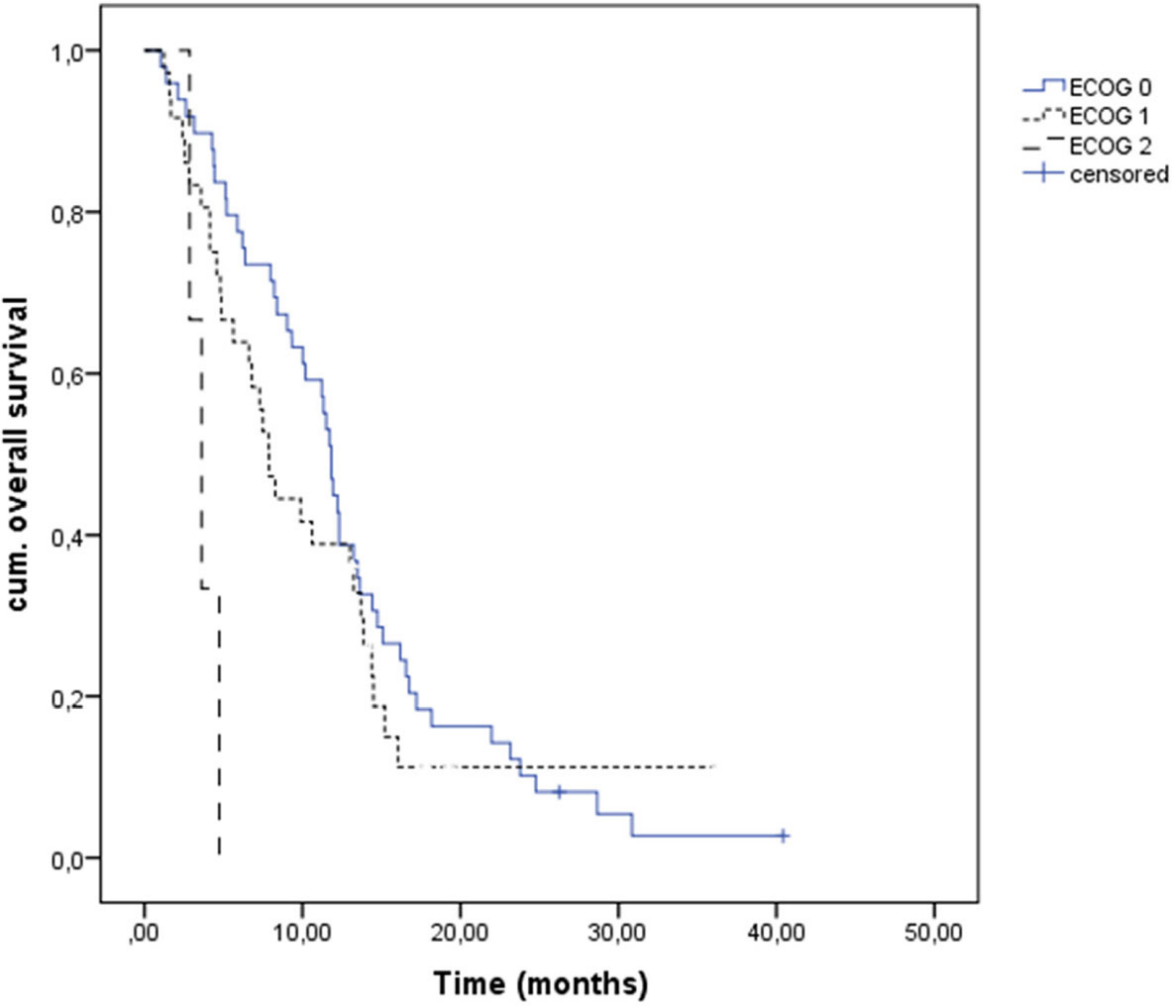
OS according to ECOG PS

Median OS in our patients was 10.2 months [95% CI 7.1;13.3], and also differed significantly between the ECOG groups with 11.8 months [95% CI 11.0;12.6] for ECOG PS 0, 7.9 months [95% CI 6.4;9.3] for ECOG PS 1 and 3.6 months [95% CI 2.4;4.8] for ECOG PS 2 (overall comparison *p* = 0.003) (Fig. 2). While dose modifications did not significantly influence OS (*p* = 0.078), patients with permanent therapy discontinuation due to toxicity lived significantly shorter (2.8 months [95% CI 2.3;3.3] vs. 11.5 months [95% CI 9.6;13.4], *p* < 0.001). OS did not significantly differ between the groups of patients with metastasized compared to locally advanced tumors or for different tumor localizations.

### Comparison of different age groups

When we compared older patients (≥ 65 years) and younger patients, we did not find significant differences in frequency of therapy interruptions, dosage modifications, or appearance of any toxicity CTCAE grade 3 or higher (Table 2). An age ≥ 65 years was not associated with significantly different PFS or OS. Median OS of patients ≥65 years was 7.9 months [95% CI 5.8;10.0] compared to 11.2 months [95%CI 8.9;13.6] for patients aged younger than 65 years, but this difference was not significant (*p* = 0.83).

## Discussion

In the U.S., the median age at diagnosis of pancreatic cancer is 71 years (data for 2006–2010, seer.cancer.gov). Two-thirds of patients are 65 years and older, and 41% are even 75 years or older. However, the FOLFIRINOX trial excluded patients older than 75 years (median age 61), and the vast majority of patients was even younger than 66 years (71%, 244 out of 342). Similarly, other clinical trials establishing the standard treatments for advanced pancreatic cancer had median ages between 62 and 64 years [12, 22, 23]. Although the MPACT trial introducing nab-paclitaxel + gemcitabine did not have an age limit, median age was also 63 years (range 27–88), with 42% of patients older than 65 years and 10% of patients older than 75 years [24].

In our heterogeneous academic outpatient collective, the survival times for first-line FOLFIRINOX treatment were in good accordance with the PRODIGE 4 trial collective and other retrospective analyses [17, 18]. 57% of our patients needed dose reductions, confirming the regimen’s substantial toxicity profile. Several authors have recommended using modified FOLFIRINOX regimens with reduced doses of chemotherapy to decrease the frequency of side effects. In our cohort, survival times for patients with dose reductions were not inferior to those receiving full-dose FOLFIRINOX, supporting the thesis that dose reductions might be possible without reducing efficacy [25]. The superior PFS of our patients with protocol adjustments is probably associated with the fact that dose reductions occurred more frequently in patients with a longer treatment period, reflecting the cumulative toxicity with prolonged chemotherapy. It might be a reasonable approach to start with full-dose FOLFIRINOX but to carefully monitor side effects and quickly adapt the doses if necessary. In patients that are deemed borderline fit for FOLFIRINOX, it might be wise to immediately start with a reduced dose to avoid toxicity-induced treatment discontinuation since this seems to be associated with a worse outcome. In terms of toxicities, no data for the different age groups were reported by the PRODIGE 4 trial authors. We did not find evidence that the subgroup of patients ≥65 years that was initially deemed fit enough for FOLFIRINOX treatment had an increased incidence of toxicities. In the PRODIGE 4 trial, the subgroup analysis showed no hint for a worse survival for patients between 65 and 74 years [13] and also in our analysis, the difference in OS for the age groups did not reach statistical significance. However, the small sample size of our old patients should be noted.

For gemcitabine-based palliative regimes, we have previously found that older patients with advanced pancreatic cancer with an ECOG PS of 0 or 1 do not have an inferior outcome or more toxicities than younger patients [26]. These findings are consistent with studies in other solid malignancies [27–29]. Thus, for advanced pancreatic cancer, the feasibility and efficacy of modern palliative chemotherapy regimens seems to be independent of chronological age. Our analysis highlights the prognostic impact of the initial ECOG PS, which allows a rapid evaluation of the patients´ resources with respect to tumor-specific treatment. In the pivotal PRODIGE 4 trial, only patients with an ECOG PS of 0 or 1 were included, and the MPACT trial included less than 10% of patients with an Karnofsky-score of less than 80%. Contrasting clinical trials with very strict inclusion criteria, “real-life” analyses include patients with more comorbidities and/or a reduced general condition. However, differences in the rated ECOG score between different physicians as well as different medical disciplines have been observed [30]. There is no doubt that older patients will have on average a worse ECOG PS and more comorbidities than younger patients, but the decision to withhold FOLFIRINOX from old patients based only on chronological age would not be reasonable and reflects a form of “ageism”. Whether a more intensive comprehensive geriatric assessment will translate into a superior rating regarding the tolerability of oncological treatment remains unknown. Some authors suggest that gemcitabine + nab-paclitaxel might be the preferred option in older patients given the lower incidence of several adverse events such as diarrhea in comparison to FOLFIRINOX. However, although independent phase III trials should only be directly compared very cautiously, the median overall survival rates clearly favor the FOLFIRINOX protocol (median OS 11.1 months for FOLFIRINOX vs. 8.7 months for gemcitabine/nab-paclitaxel) and this active combination should therefore be considered as a valuable treatment option for old patients in good PS.

The main limitation of our study is the small number of older patients and the retrospective, non-randomized nature of the analysis. It seems unlikely that a new randomized study on FOLFIRINOX in older patients will formally prove the benefit compared to gemcitabine monotherapy or gemcitabine/nab-paclitaxel in this patient group, but our study could serve as an encouragement to offer FOLFIRINOX also to older patients with good performance status. Finally, larger retrospective analyses, e.g. from cancer registers, might put our conclusions on a more solid fundament.

## Conclusion

Our single-center experience confirms the FOLFIRINOX protocol being associated with a high rate of substantial side effects requiring dose reductions in more than half of the patients. We find some evidence, that dose reductions are possible without reducing clinical efficacy. Additionally, FOLFIRINOX seems to be a safe and efficient regimen for selected old patients with a good ECOG PS. It should not be withhold from patients solely based on the chronological age, avoiding any form of “ageism”.

## Abbreviations

CTCAE: Common terminology criteria for adverse events
ECOG PS: Eastern Cooperative Oncology Group performance status
NCT: National Center for Tumor Diseases
OS: Overall survival
PFS: Progression-free survival
RECIST: Response evaluation criteria in solid tumors
